# Exploring Stakeholder Requirements to Enable Research and Development of Artificial Intelligence Algorithms in a Hospital-Based Generic Infrastructure: Results of a Multistep Mixed Methods Study

**DOI:** 10.2196/43958

**Published:** 2023-04-18

**Authors:** Lina Weinert, Maximilian Klass, Gerd Schneider, Oliver Heinze

**Affiliations:** 1 Institute of Medical Informatics Heidelberg University Hospital Heidelberg Germany; 2 Section for Translational Health Economics Department for Conservative Dentistry Heidelberg University Hospital Heidelberg Germany

**Keywords:** artificial intelligence, requirements analysis, mixed-methods, data availability, qualitative research

## Abstract

**Background:**

Legal, controlled, and regulated access to high-quality data from academic hospitals currently poses a barrier to the development and testing of new artificial intelligence (AI) algorithms. To overcome this barrier, the German Federal Ministry of Health supports the “pAItient” (Protected Artificial Intelligence Innovation Environment for Patient Oriented Digital Health Solutions for developing, testing and evidence-based evaluation of clinical value) project, with the goal to establish an AI Innovation Environment at the Heidelberg University Hospital, Germany. It is designed as a proof-of-concept extension to the preexisting Medical Data Integration Center.

**Objective:**

The first part of the pAItient project aims to explore stakeholders’ requirements for developing AI in partnership with an academic hospital and granting AI experts access to anonymized personal health data.

**Methods:**

We designed a multistep mixed methods approach. First, researchers and employees from stakeholder organizations were invited to participate in semistructured interviews. In the following step, questionnaires were developed based on the participants’ answers and distributed among the stakeholders’ organizations. In addition, patients and physicians were interviewed.

**Results:**

The identified requirements covered a wide range and were conflicting sometimes. Relevant patient requirements included adequate provision of necessary information for data use, clear medical objective of the research and development activities, trustworthiness of the organization collecting the patient data, and data should not be reidentifiable. Requirements of AI researchers and developers encompassed contact with clinical users, an acceptable user interface (UI) for shared data platforms, stable connection to the planned infrastructure, relevant use cases, and assistance in dealing with data privacy regulations. In a next step, a requirements model was developed, which depicts the identified requirements in different layers. This developed model will be used to communicate stakeholder requirements within the pAItient project consortium.

**Conclusions:**

The study led to the identification of necessary requirements for the development, testing, and validation of AI applications within a hospital-based generic infrastructure. A requirements model was developed, which will inform the next steps in the development of an AI innovation environment at our institution. Results from our study replicate previous findings from other contexts and will add to the emerging discussion on the use of routine medical data for the development of AI applications.

**International Registered Report Identifier (IRRID):**

RR2-10.2196/42208

## Introduction

### Background

Considering the current and future challenges in health care, such as lack of health care workers and global health, artificial intelligence (AI) is regarded as one possible part of a solution to address these problems [1-4]. While there are many subspecialties of AI, for the purpose of this research we will regard AI as a general-purpose technology and follow the definition by He et al [5], who defined AI as “a branch of applied computer science wherein computer algorithms are trained to perform tasks typically associated with human intelligence” [5].

The topic of AI in health care has recently received a significant amount of attention in research, policy making, and in the general population [6-8]. However, the interest and attention so far have not led to the expected numbers of development and implementation activities in Europe, which have been reported by the Joint Research Centre of the European Commission [9] and in original research [10,11]. This gap has been attributed, in part, to low availability of large and high-quality data sets that are necessary for the development of AI tools [12,13]. Health care institutions inherently store large quantities of these data sets, but access can be challenging due to legal and ethical regulations [12,13]. In addition, legal uncertainty surrounding the (partly) automized testing of routine medical data against defined gold standards persists [14].

Another uncertainty pertains to the perspective of patients regarding the use of their medical data for AI development. Aitken et al [15] conducted a systematic review on the question of public attitudes to data sharing for health research and synthesized findings from qualitative research. They identified an overall widespread conditional support, with conditions including the assurance of the individual’s confidentiality, a preference for anonymity of data, and assurances of data security. The applicability of these findings to AI as a fundamentally new general-purpose technology was examined in 2 studies by McCradden et al [16,17]. The first study among a sample of the general population revealed mostly negative views about AI. Participants in this first study considered consent, transparency of AI use, and assurances of data privacy as important conditions for supporting the use of health data for AI research [17]. In 2020, McCradden et al [16] studied a similar question among a sample of patients with meningioma, caregivers, and health care providers in Canada. Participants recognized the high value of health data as an important resource for medical research. Again, consent emerged as one of the most important conditions for support. Yet, participants in their study also discussed scenarios in which consent could be bypassed [16].

It is plausible to assume that the preferences and opinions derived from previous research could vary between different cultural contexts and between the general population and current patients. To the authors’ knowledge, there are no previously reported findings on patient perceptions of data usage for AI research in Germany.

### Developing a Hospital-Based Generic Infrastructure to Enable Research and Development of AI Algorithms

The aforementioned questions are addressed by the *pAItient* project (Protected Artificial Intelligence Innovation Environment for Patient Oriented Digital Health Solutions for developing, testing and evidence-based evaluation of clinical value), which has the objective to establish an AI innovation environment as a proof-of-concept extension of the already existing Medical Data Integration Center [18,19] at the Heidelberg University Hospital [20]. Project partners include the German Cancer Research Center (DKFZ), the German Research Center for Artificial Intelligence (DFKI), and MINT Medical GmbH (Heidelberg, Germany) [20].

Within the project, knowledge of the legal requirements and needs stakeholders such as patients, health care providers, and industry partners have toward the planned infrastructure was defined as an important antecedent. We propose that a comprehensive analysis of stakeholder and legal requirements will build the necessary foundation for a high acceptance and usefulness of the planned infrastructure. Although patients are not going to be direct users or beneficiaries of our planned infrastructure, it was decided to include a sample of patients from our institution as an additional stakeholder group. To facilitate later discussions on patient and public involvement in research, the Guidance for Reporting Involvement of Patients and the Public—short form (GRIPP2-SF) [21] framework will be used (Multimedia Appendix 1). Later stages of the *pAItient* project encompass the development of the necessary information technology (IT) architecture concepts, the deployment of said infrastructure, and the application of medical use cases to test the infrastructure. This final step will be accompanied by an evaluation studying the effects of the AI innovation environment on patients and health care providers.

This project is funded by the German Federal Ministry for Health.

## Methods

### Study Design

This is a multistep mixed methods study of the opinions of different stakeholders who may be involved in the planned AI innovation environment. In a first step, semistructured interviews were used to gain a fundamental understanding of (1) the requirements participants had to be able work with an AI innovation environment at an academic hospital and (2) the requirements that must be met in order for patients to allow for the use of their data within an AI innovation environment.

Findings from the interviews enabled the tailoring of survey items to the specific participant group for the second part of the study.

Legal requirements for the AI innovation environment were deduced from relevant literature.

### Study Population

The study population was recruited from 6 different stakeholder groups: (1) researchers from a biomedical research institute, (2) researchers from an AI research institute, (3) employees from start-up companies in the field of AI development, (4) employees from an AI imaging company, (5) patients at the Heidelberg University Hospital, and (6) physicians actively working in inpatient or outpatient health care in Germany.

The detailed inclusion and exclusion criteria can be found in the study protocol [20]. As shorthand and due to overlaps in methodology and content, groups 1-4 will be referred to as “professional groups.” “Patients” will be used as shorthand for group 5. A detailed description of the methodology for the group of physicians (group 6) has been published elsewhere [22] and will only be explained in a cursory manner for the purpose of this work.

### Recruitment and Sampling

A convenience sampling strategy was chosen. Potential participants from groups 1-4 and 6 were recruited through the project network and known contacts. Invitations to participate in either the interviews or the survey were sent via email.

Patients were recruited through a purposive sampling approach in different departments at the Heidelberg University Hospital (Department of Obstetrics and Gynecology, Department of Internal Medicine, outpatient department at the National Center for Tumor Diseases Heidelberg) where they were approached by a member of the study team and invited to participate. To recruit patients without the physical presence of a study team member, leaflets containing information on the study and contact data of the study team were left in appropriate spots at the participating clinical departments.

All participants received verbal and written information about data protection regulations and study procedures and aims. They were given adequate time to ask questions and were asked to return a signed letter of intent before participating in the study. As no personal data were collected in the later survey, participants did not have to sign a letter of intent for this part of the study. Participants were not offered reimbursement.

### Data Collection

Interviews were conducted via web-conferencing tools (groups 1-4) or via telephone (groups 5 and 6). Interviews with patients were conducted by LW, a female researcher who has a background in health services research and experience with qualitative interviewing. Interviews with physicians were conducted by another research group from our institution [22]. Interviews with the professional groups were facilitated by LW (eg, taking notes, technical organization) and conducted by OH, a male researcher who has a background in medical informatics and received a training in qualitative interviewing. No prior relationship was established with the interviewees. Nonparticipants were not present during the interviews. No repeat interviews were carried out. Field notes were taken. Transcripts were not returned to participants.

All interviews were audio-recorded and later transcribed verbatim. All interviews were based on group-specific, semistructured interview guides. A detailed description of the interview guides’ development can be found in the study protocol [20]. Translated versions of the interview guides can be found in Multimedia Appendix 2 (groups 1-4 and 6) and in the previously published article on the physicians’ interviews within the *pAItient* project [22].

Prior to the interviews, participants were asked to fill in a short sociodemographic questionnaire.

Within an interprofessional team of health services researchers and medical informaticians (LW, GS, and OH), the base structure of the survey for the second part of the study was agreed upon. While the questions were the same for groups 1-4, the items that should be rated or answered were adapted to the findings from the interviews from each group. The developed survey was piloted and discussed in an online session with other medical informaticians and AI researchers whereafter small changes were made to the wording. In the first section, the survey asked participants to name regularly used software and hardware for the development of AI tools, their institution’s goals, and what kind of data are necessary. The second section asked participants to reflect on their experiences with academic hospitals as data providers (eg, advantages and disadvantages or bad experiences with these kinds of cooperation). The third section asked participants to rate possibilities for academic hospitals to improve and support cooperation in AI development.

REDCap (Research Electronic Data Capture) hosted at the Heidelberg University Hospital was used for study data collection and management. REDCap is a secure, web-based software platform designed to support data capture for research studies [23,24].

### Data Analysis

Qualitative data from groups 1 and 5 were transcribed and analyzed in MAXQDA 2020 (VERBI GmbH), following a thematic analysis approach (for the analysis of physician interviews, please see Kamradt et al [22]). LW coded the data. First, themes were identified deductively from the interview guide and inductively from the present data. The resulting coding scheme was combined with the interview notes from groups 2-4 and discussed in an interprofessional group of health services researchers and medical informaticians. Participants did not provide feedback on the findings. As soon as consensus on the most relevant aspects for the survey for each group was agreed upon, the survey for groups 1-4 was designed. A translated version of the developed survey is provided in Multimedia Appendix 3.

Data from the filled-in surveys and sociodemographic questionnaires were analyzed descriptively (eg, means and SDs) using Microsoft Excel. Answers to open-ended questions were reviewed, paraphrased, and grouped.

### Ethics Approval

This study was approved by the Ethics Committee of the Heidelberg University Hospital (approval number S-241/2021) in March 2021.

## Results

### Overview

In the following section, results of the qualitative interviews and the survey will be presented. Reporting of the qualitative findings and procedures follows the Consolidated Criteria for Reporting Qualitative Research (COREQ) guideline [25] (Multimedia Appendix 4). First, themes from the qualitative interviews with groups 1-4 will be explored and illustrated with quotes and corresponding survey results.

In the second part, themes derived from the interviews with patients will be introduced and illustrated with relevant quotes as well.

All direct quotes were translated from German with due diligence.

Results from the interviews with physicians have been published by Kamradt et al [22].

### Professional Groups (Groups 1-4)

#### Participant Characteristics

We successfully recruited 8 interviewees working in a biomedical research institution (n=3), an AI research institution (n=1), a software developing company (n=2), and in AI-focused software start-up companies (n=2). The mean length of the audiotaped interviews was 47 minutes.

Concerning the quantitative measures, we collected 17 surveys from the different professional stakeholder groups.

#### Goals

To characterize the participating institutions, interviewees were asked about possible goals motivating their work or their institution. During the interviews, participants highly valued the aim to achieve tangible benefits for patients and the hospitals they are working with. These benefits should be achieved through high-quality research that is able to be implemented in real-world settings. All participants in the interviews rated patents at a lower importance.

For me as a scientist, I don’t really care about patents […]. Personally, I would much rather like to see a result for the patient or in patient care.

PG1_1, Transcript position 68

In the anonymous survey, participants from groups 1 and 3 rated patents as one of their second lowest or least important goal, respectively. Interview participants from private companies emphasized achieving business goals (eg, successful product development).

Survey participants were asked to rate their approval to the goals derived from the interviews on a slider ranging from 0% to 100%. Table 1 lists and compares these goals.

**Table 1:** Survey results—goals and rating of agreement with these goals (N=17).^a^

Goals	Biomedical AI^b^ researchers	AI research institution	Software developing companies
Founding of new companies/spin-off companies	36 (0-71)	N/A^c^	N/A
Development of patents	49 (0-72)	N/A	26 (7-55)
Benefit for patients and patient care	83 (78-88)	83 (61-94)	N/A
Achieving research goals (eg, publications, presentations at conferences)	84 (73-100)	89 (69-100)	N/A
Successful product development	N/A	N/A	90 (81-100)

^a^Data are presented as mean rate of approval (in percentage) (range).

^b^AI: artificial intelligence.

^c^N/A refers to items that were not asked in the respective group.

#### Intellectual Property

All statements concerning intellectual property (IP) were assigned to this theme. IP was seen as a risk that has to be managed to be able to access data.

I think at the moment – this topic [IP] is not as urgent, because lack of data is such a big problem and many AI-developers will take this risk, many risks, to access data.

PG1_2, TP28

In general, interviewees had the opinion that the institution that designed the algorithm is entitled to IP. They were in favor of regulating these questions via contracts with the institutions providing the data. Considering contracts, licensing options were viewed unfavorably because they provide only temporary access. While IP was a topic of lower importance, differing IP regulations were seen as a measure to compare data-providing institutions with each other.

#### Data Processing

Another aspect that was discussed in the interviews concerned data processing and in what kind of IT infrastructure the data derived from the hospital should be analyzed, trained, and validated. Researchers from group 1 highly favored having the data easily accessible within their own infrastructure. Yet, they were also accepting of the idea to access data through remote access options. Necessary antecedents for this approach were a good UI and a stable connection. Advantages of this option were the ability to view the data within their original system architecture and that no exchange of sensitive patient data would be necessary.

If that works well, the UI, the stability – and you can also get enough information about the data, so you are able to decide how to build your model. In that case, I think that makes sense and it would also make a lot of things easier, because you don’t have to exchange data all the time.

PG1_3, TP30

A possible disadvantage was the risk that a hospital’s technical infrastructure could lack the computing power to train algorithms. To counter this risk, 1 participant suggested to use a smaller subset of the data outside the hospital system and to conduct the validation within the hospital system.

#### Necessary Hardware/Software

Survey participants were asked to indicate if they use or do not use specific hardware and software for the development of AI tools. The results of this question are presented in Multimedia Appendix 5.

#### Data Used for the Development of AI Tools

This question asked interview participants to indicate and explain which data or type of data they typically use for their development processes. They stated that they would ideally work with annotated data and that the preceding annotation workflow should be made transparent to them. The availability of data with high-quality annotation reduces the amount of data needed. Concerning specific use cases, they would also like to access data other than patient data/medical records, for example, data from surgical instruments (eg, endoscopes, data from anesthesiology machines) and data from external sources (eg, opening and closing times of operation room doors).

Survey participants were asked to check a box if they typically worked with these kinds of data. Table 2 lists and compares types of data across the different groups.

**Table 2:** Survey results—types of data typically used for the development of AI^a^ tools (N=17).

Types of data used for the development of AI tools	Biomedical AI researchers^b^, n/N (%)	AI research institution^b^, n/N (%)	Software developing companies^b^, n/N (%)
Imaging data	4/4 (100)	8/9 (89)	4/4 (100)
Structured electronic health record data	4/4 (100)	N/A^c^	N/A
Unstructured electronic health record data	1/4 (25)	N/A	N/A
Text data with context information	3/4 (75)	7/9 (78)	1/4 (25)
External data (eg, terminologies such as ICD-10^d^, SNOMED^e^)	3/4 (75)	4/9 (44)	1/4 (25)
External sources (eg, local treatment guidelines)	4/4 (100)	N/A	N/A
Data from medical instruments (eg, intraoperative endoscopy)	3/4 (75)	N/A	N/A
Data from nonmedical peripheral sensors (eg, operation room doors)	2/4 (50)	N/A	N/A
Ground truth/characterized/annotated data	4/4 (100)	N/A	N/A

^a^AI: artificial intelligence.

^b^Total participants in agreements and sample percentages.

^c^N/A refers to items that were not asked in the respective group.

^d^ICD-10: 10th revision of the International Statistical Classification of Diseases and Related Health Problems.

^e^SNOMED: Systematized Nomenclature Of Medicine.

#### Advantages and Disadvantages of Working With Academic Hospitals

A further aspect that was discussed with interview participants was the cooperation with academic hospitals and previously experienced or anticipated advantages and disadvantages of this cooperation. Possible advantages that were mentioned included the availability of high amounts of data, special data (eg, rare diseases), the possibility to test and validate algorithms within a protected environment, and the hospital’s willingness to invest additional effort in research projects. Another advantage was the proximity to a “real environment” in a hospital setting:

So [from working with an academic hospital] you don’t only get artificial data or only specific data from special cases but the kind of cases that typically reoccur in a hospital. So, they are from a “real environment”. And the closer you can get to the actual environment, the easier you can develop an application that is able to deliver good results.

PG1_3, TP24

Survey participants were asked to rate their agreement with the mentioned advantage on a 5-point Likert scale (0=I do not agree at all, 4=I completely agree). Table 3 lists and compares types of possible or previously experienced advantages across the different groups.

**Table 3:** Survey results—agreement with statements on advantages of cooperating with an academic hospital (N=4).

Possible or previously experienced advantages	Biomedical artificial intelligence researchers^a,b^, n/N (%)^c^
Data from a “real environment”	4/4 (100)
Personal contact with future users of the developed artificial intelligence tools	2/4 (50)
Simplified identification of potential use cases	2/4 (50)
Extensive technical facilities	1/4 (25)
Scaling effects	1/4 (25)
Many potential clinical partners	0/4 (0)
High degree of professionalization	1/4 (25)
Hospital takes on administrative tasks	0/4 (0)
Availability of contact person in case of queries in the data	0/4 (0)

^a^This question was only presented to participants who stated that they have previously worked with academic hospitals. Because of the small sample size and the resulting risk of reidentification, answers from groups other than group 1 cannot be reported.

^b^Total participants in agreement and sample percentages.

^c^Responses of “agree” and “rather agree” were grouped together.

Besides advantages, potential disadvantages of these cooperations were discussed. Interview participants described bad experiences with extensive bureaucracy, which made getting access to data difficult. Administrative contact persons in the hospitals that they had encountered previously were remembered as anxious to violate data privacy regulations. This observation, in combination with lacking technical knowledge to understand the research proposals, has previously led to uncertainties, delays, and to a “play it safe” approach regarding research ideas.

And the technologies are getting more and more complex. And this uncertainty, which is understandable, can result in a tendency to “play it safe”. And this can, of course, lead to problems or exhausting processes, at least from a researchers’ perspective, who are always like “this is great, let’s get started immediately.

PG1_2, Pos. 32

Sometimes, data sets they had received were not self-evident and consultations with hospital physicians were necessary to work with the data. In addition, data sets they had been offered were hard to use and not in the ideal format (eg, unstructured electronic health record or system data). Interviewees also mentioned that the necessity to work within a research study setting introduced further difficulties, such as having to get approval from the ethics committees. Another risk that has to be managed is “overfitting,” which could result from a too strong reliance on data from 1 source.

Survey participants were asked to rate their agreement with the mentioned disadvantage on a 5-point Likert scale (0=I do not agree at all, 4=I completely agree). Table 4 lists and compares possible or previously experienced disadvantages across the different groups.

**Table 4:** Survey results—agreement with statements on advantages of cooperating with an academic hospital (N=4).

Possible or previously experienced disadvantages	Biomedical artificial intelligence researchers^a,b^, n/N (%)^c^
Complex legal requirements concerning data privacy and other regulations	4/4 (100)
Complex bureaucracy	4/4 (100)
Diversity and number of contact persons	4/4 (100)
Requirement to work within research studies	1/4 (25)
Overfitting of a developed algorithm to 1 hospital	3/4 (75)
Legal and regulatory fears among (hospital) administrative staff	4/4 (100)
Decision makers not competent to understand complex innovations and their implications	3/4 (75)
Discussions about intellectual property	3/4 (75)
Tools are too complex	0/4 (0)
Launching new projects is cumbersome	3/4 (75)
Data quality not adequate	4/4 (100)

^a^This question was only presented to participants who stated that they have previously worked with academic hospitals. Because of the small sample size and the resulting risk of reidentification, answers from groups other than group 1 cannot be reported.

^b^Total participants in agreement and sample percentages.

^c^Responses of “agree” and “rather agree” were grouped together.

#### Potential Areas for Support

This code was assigned to all statements concerning potential areas where academic hospitals could support the participants’ institutions. Interview participants suggested the installation of a platform enabling a quick overview over which algorithms had already been developed or tested at the hospital in question, which groups are working on projects related to AI, and which data are available and could be used for the development of AI tools. The interviewees argued that a platform like this could improve cooperation, innovation, and creativity.

To establish an environment to look at data, meta data – to get a good insight how the data are available, what you can do with them. […] And you should also be able to see what has already been done, for example, what are the parameters for a deep leaning model somebody has set up. […] I know this is not easy. But that is how I think a platform like this should work, it should enable a lot of exchange between researchers. […] This is how you can find new partners for cooperation. You can easily see which group is developing new expertise in which area. And so you can find new people easier.

PG1_3, TP36

Interviewees also suggested a closer cooperation with clinicians, for example, in finding common goals. Here, clinicians could give more insight into which algorithms or software solutions could make their work easier. Clinicians could also support in the evaluation of newly developed tools to determine whether these are usable and beneficial for clinical practice. At the same time, interviewees would also like to be able to explore data independently. This independent exploration should be supported by platforms with a good UI and characteristics about the provided data should be communicated clearly (eg, quantity, quality, annotations, ground truth). In this context, participants suggested the introduction of new tools into the physicians’ workflow, which will allow for the parallel annotation of collected data.

Survey participants were asked about their perceived usefulness on a 5-point Likert scale (0=not helpful at all, 4=very helpful). Table 5 lists and compares different possible supportive measures an academic hospital could introduce to support AI developers.

**Table 5:** Survey results—possible supportive measures and their evaluation regarding usefulness (N=17).

Possible supportive measures and their evaluation regarding usefulness	Biomedical artificial intelligence researchers^a^, n/N (%)^b^	Artificial intelligence research institution, n/N (%)^b^	Software developing companies, n/N (%)^b^
Simplified, standardized process for data privacy processes	4/4 (100)	N/A^c^	4/4 (100)
Establish or simplify contact to clinical users	4/4 (100)	N/A	4/4 (100)
Provide insight into clinical processes	4/4 (100)	N/A	N/A
Platform offering an overview of previously developed algorithms	3/4 (75)	N/A	N/A
Platform offering an overview of available data	4/4 (100)	N/A	N/A
Platform offering an overview of potential partners for cooperation	4/4 (100)	N/A	N/A
Data from medical instruments (eg, intraoperative endoscopy)	3/4 (75)	N/A	N/A
Transparent communication about character and quality of available data	4/4 (100)	N/A	N/A
Provision of annotated data	4/4 (100)	N/A	N/A
Enabling of federated learning	3/4 (75)	N/A	N/A
Combination of electronic health record and research database	3/4 (75)	N/A	N/A
More opportunities to apply or test developed tools in a hospital environment	3/4 (75)	N/A	N/A
Finding research questions/medical use cases in a collective approach with clinicians	N/A	9/9 (100)	N/A
Protection from and clarification of liability issues	N/A	9/9 (100)	N/A

^a^Total participants in agreements and sample percentages.

^b^Responses of “agree” and “rather agree” were grouped together.

^c^N/A refers to items that were not asked in the respective group.

### Patients (Group 6)

#### Participant Characteristics

A total of 6 patients were recruited successfully. One patient withdrew their consent after the interview. Interviewees were asked to fill in a sociodemographic questionnaire. Table 6 provides information on the participant characteristics. The interview duration varied between 19 and 36 minutes (mean 28 minutes).

Following the thematic analysis [26] approach, 5 main themes were identified in the material and will be presented in the subsequent paragraphs along with supporting direct quotes.

**Table 6:** Participant characteristics (N=5).

Characteristics		Participants
**Gender, n (%)**		
	Female	4 (80)
	Male	1 (20)
Age in years, mean (SD); range		54.4 (8.3); 39-62
**Education, n (%)**		
	High school diploma	4 (80)
	Intermediate secondary education	1 (20)
**Chronic or current disease^a^**		
	Breast cancer	4 (80)
	Asthma	2 (40)
	Diabetes type 2	1 (20)
	Psoriasis	1 (20)
Number of doctor’s visits or hospital stays in the past 3 months, mean (SD); range		6.6 (3); 3-12
Affinity toward modern technologies^b^, mean (SD)		3.8 (0.7); 3-5

^a^Multiple answers possible.

^b^From 1 (very low) to 5 (very high).

#### Reflections on AI as a New Technology

This code was assigned to all statements concerning AI technologies in general, such as previous knowledge, hopes, and fears. Patients in this sample had high hopes of AI possibly helping in the treatment of their own disease in the future and thus were optimistic about their participation in the study and its aims.

I am happy to see that research in this area is happening here. It is tangible. [...] That is also one of the reasons why I chose to participate in this study, I am curious.

PG5_2, TP56

Although none of the participants in this study said that they themselves had fears or negative perceptions surrounding AI, they recognized the prevalence of these fears within the society:

A lot of people have this negative image in their head. Robots are taking over, mankind can’t do anything anymore and is reigned by AI, by a machine. And this image creates fear. That is understandable. […] But I am very positive towards robots and AI.

PG5_1, TP30

The interviewees also discussed that these fears and a lack of understanding of AI could lead to misunderstandings and low willingness among other patients to agree to the use of their data.

#### Data Sharing

The patients in this sample were also optimistic about the potential use of their medical data for the development of AI tools and mentioned several potential positive effects. Again, they saw a potential to support research and treatment in their disease.

I think my treatment data could maybe help patients, who will be affected by the same disease later. So it could help in the actual treatment of this disease.

PG5_5, TP8

I have stage IV breast cancer and would share as much data as necessary. I would have no problem at all to share personal data or health data and so on. I would see that as an opportunity.

PG5_4, TP12

Concerning the kind of data that can be shared, they only noted that personal data such as name, phone number, and email address should not be shared. They emphasized that medical data should be shared in a form which does not allow for reidentification. Participants further demanded to be informed about what will happen with the data they shared, why their data will be necessary, and what other organizations will be involved. In general, they supported an option to limit the kind of data that can be shared. However, they also argued that many other patients could lack the necessary knowledge and information to judge these issues. In this context, they also worried that physicians or other contact persons might not have enough time to explain the data-sharing concepts to all their patients, especially patients with lesser previous knowledge.

The lack of time for necessary explanations was also mentioned as a reason why the patients in our sample would potentially refuse to share their data. Other reasons included, for example, if there is a risk of reidentification of their data, if the benefit for medical research is not stated clearly, and if the reputation or trustworthiness of the institution they are being treated at is bad.

#### Cooperation With Other Organizations

Generally, participants highly valued and trusted the existing data privacy regulations in public institutions. Yet, patients were sceptic toward the involvement of too many different organizations. This was explained by a perceived higher risk of data leaks or misuse when too many players are involved. Skepticism was especially notable toward private companies. Here, participants clearly differentiated between private companies and publicly funded institutions:

Public institutions are good. But as soon as private companies are involved, I would like to have transparency and would like to know which companies.

PG5_4, TP47

On the other side, interviewees stated that having a bigger group with several different organizations also means that research could be done faster and better due to more researchers working on the same questions.

#### Consent Process

Concerning the consent process for data sharing, patients preferred to have a conversation with their physician in which the physician would take time to inform them about the data-sharing process and answer potential questions. In general, the person conducting the consent process should be trustworthy and well informed. Brief, written information should be handed out beforehand, so that patients are able to prepare questions. Participants demanded to be informed about which companies are involved, a very broad overview over what will be done with their data, and how their data will be protected.

There has to be a relationship of trust with this person. It is not really relevant whether that is a senior or junior physician or research associate, it just has to be made clear what will happen. And another thing would be important for me – will the data only go to academic hospitals or will they also be transferred to pharmaceutical companies? And under which conditions?

PG5_3, TP48

#### Requirements for Consent to Data Sharing for AI Development

Over the course of the individual interviews, the requirements for consent to data sharing for AI development emerged. Participants stated that they needed to be able to retract their consent. The institution they are sharing their data with has to be trustworthy and their data should not be reidentifiable. If private companies are involved, a medical benefit for patients should be the objective. Finally, patients demanded to receive transparent and comprehensive information beforehand.

### Legal Requirements

The General Data Protection Regulation (GDPR) was introduced in 2018 by the European Parliament and Council of the European Union [27]. It includes protections of fundamental rights of data subjects (e.g., personal data of European Union citizens). One of these protections refers to the transparency of automatic decision-making systems, granting data subjects distinctive rights when their data are processed by these systems [28]. For example, data subjects should be informed about the existence of automated decision-making processes, they have the right to not to be subject to decisions solely based on automated processing, and they have the right to obtain human intervention [28,29]. On a technical level, this “right to explanation” [28] hence demands a certain level of technical explainability to realize these protections for European Union citizens.

### Synthesis

**Figure 1:**
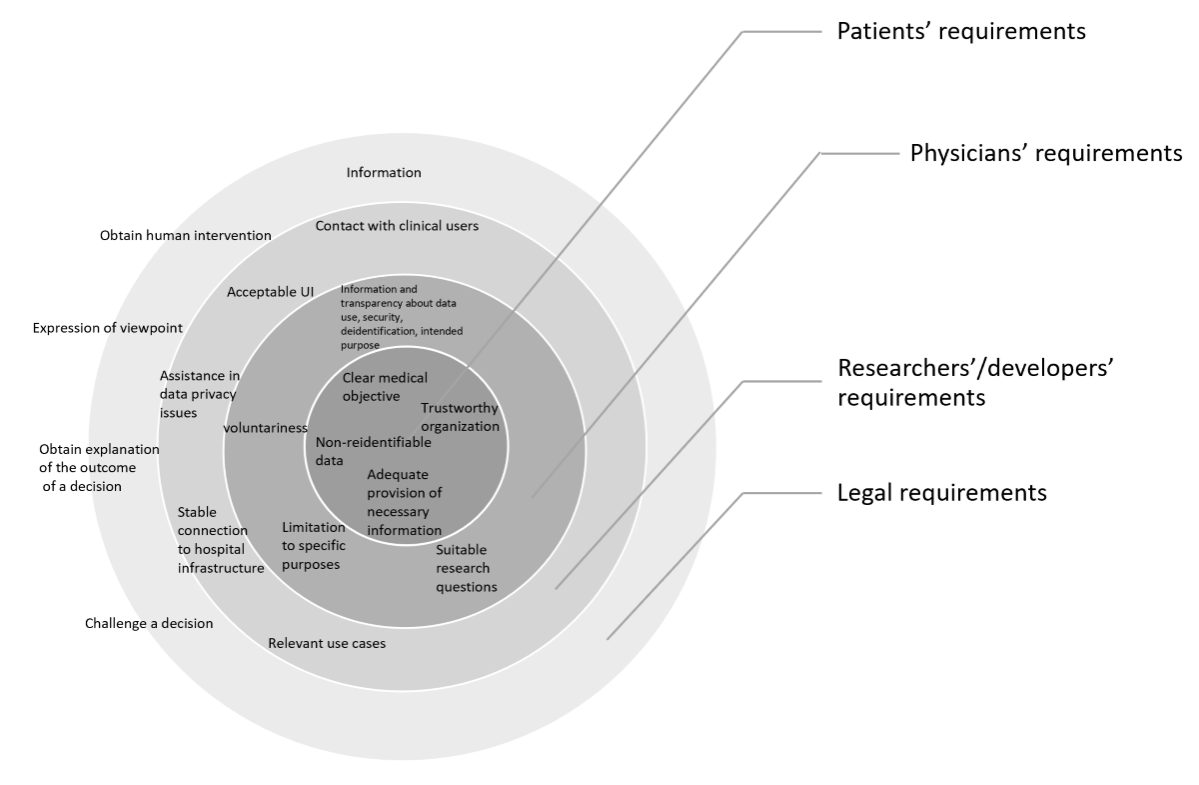
Developed requirement model for the planned pAItient infrastructure. pAItient: Protected Artificial Intelligence Innovation Environment for Patient Oriented Digital Health Solutions for developing, testing and evidence-based evaluation of clinical value; UI: user interface.

To merge the findings from all the studied participant groups within the *pAItient* project, the results from this study were combined with the findings from participant group 6 (physicians), which were reported by Kamradt et al [22]. In addition, legal requirements were deduced from the literature. To visualize these combined findings, a requirements model was developed (Figure 1). Patients and their requirements were placed in the center of the model, as data from their treatment, diagnostic measures, and hospital stay will be collected. Physicians, who are responsible for data collection and entry, were placed in the next layer. Researchers and developers represent external stakeholders, and are thus placed on an outer layer. As legal requirements influence the actions of all stakeholders, they were placed on the outermost layer.

This model was communicated to all project members. The requirements were operationalized and items relevant to the development of the IT architecture were implemented in the IT concept. This guarantees consideration of these requirements in the long term of the AI innovation environment.

## Discussion

### Principal Findings

The aim of this study was to collect the requirements for the development of AI-based algorithms based on routine medical data within a hospital-based generic infrastructure. Views from different stakeholder perspectives were included in this study, such as patients, physicians, AI researchers, and industry employees. Legal requirements were deduced from the literature. The identified requirements covered a wide range and were inherently conflicting sometimes, even within the stakeholder groups. However, a requirement model was developed, which depicts the identified requirements in 4 different layers. In the center, the most relevant patient requirements for data use were listed (adequate provision of necessary information for data use; clear medical objective of the research and development activities; trustworthiness of the organization collecting the patient data; and data should not be reidentifiable). The second layer represents the physicians’ requirements for participating in AI research and development projects (voluntariness; information and transparency about data use, security, deidentification, and intended purpose; nonprofit use; suitable research questions; and limitations to specific purposes) [22]. The third layer represents the requirements AI researchers and developers formulated for working with routine medical data within the planned infrastructure (contact with clinical users, acceptable UI for shared data platforms, stable connection to the planned infrastructure, relevant use cases, and assistance in dealing with data privacy regulations). The outermost layer depicts the legal requirements derived from the GDPR (right to information, right to obtain human intervention, right to express their point of view, right to obtain an explanation of the outcome of a decision, right to challenge a decision) [27,28]. This developed model will be used to communicate stakeholder requirements within the *pAItient* project consortium.

### Comparison With Prior Work

Comparable projects to build infrastructures and networks enabling AI development are ongoing, such as the CHAIMELEON project [12]. However, to the authors’ knowledge, AI researchers’ and developers’ requirements for working with hospital-based generic infrastructures have not been reported in the literature. Thus, our findings from these groups could inform both ongoing projects and hospitals aiming to build comparable infrastructures to collaborate in the future and to use synergies.

Concerning the findings from interviews with patients, previously reported requirements could be replicated in our sample. McCradden et al [16] conducted a qualitative study among a sample of patients with meningioma in Canada, identifying requirements such as consent, trust in health care organizations, and privacy. These requirements also emerged in discussions with patients from the study presented in this paper, although patients were recruited from a different cultural context and were affected by a wider range of diseases. Skepticism toward the involvement of private or for-profit institutions has also been identified in earlier research, whereas these previous findings have been limited to non-AI–specific data use [30-33]. However, our formative findings indicate that the concept of this skepticism is transferable to data use for AI development.

The importance of respecting patient requirements for data use in AI development is evident, as many studies have shown that patients’ wishes and expectations for the use of their data in health research can differ from researchers’ wishes [31]. Tosoni et al [31] formulated an “unfortunate chasm between knowledge and practice” regarding the use of medical data for research from the perspectives of health care institutions, researchers/developers, and patients. While both literature and our findings advocate for a more active role of patients with regard to informed consent for data use, lack of knowledge can also impede with the patients’ ability to give informed consent for data use. As one of the patients in our study noted, it can be difficult for patients to understand the presented research purposes. This difficulty can make it harder for them to ask the necessary questions and could even lead to a refusal of consent for data use. Patients can also lack the necessary terminology and understanding of data processing steps, which some argue is currently an inherent issue with AI technologies [34]. In this context, they are described as “black boxes,” machines with nontransparent workings. The GDPR regulates a certain level of AI explainability [28], an aspect which was also ranked highly in a choice-based survey in the general population in Denmark [35]. In our sample, patients did not mention this aspect. Still, they requested both the ability to retract consent at any time and the complete deidentification of their data. However, these requirements cannot be met simultaneously. The high trust patients have in public institutions as well as the enthusiasm to support medical research with their data can build an excellent foundation for trusted partnerships with health care institutions, health care providers, and AI researchers and developers. To foster these partnerships and establish an “equal playing field” for patients, they should be given necessary information within a comfortable and open setting, empowering them to make the right decisions for themselves and their medical data.

### Strengths and Limitations

A limitation for this study is the relatively small sample size. Concerning groups 1-4, this could be explained by a lack of time of potential participants due to a high number of concurrent projects and research activities. To increase the number of potential participants, the survey was translated into English. This led to an improvement in response numbers. The survey results were very homogenous, with mostly high rates of approval. This could imply that the interview participants represented their institutions well, indicating that the results from these groups are plausible despite the small sample size. Nonetheless, the possibility of missing potentially relevant aspects cannot be disregarded.

Recruiting patients for the interviews was challenging as well. This could be explained by the complexity of the topics of AI and data use, resulting in a low interest in participation in research [20]. This has been identified as a barrier before [36]. To improve recruitment in our study, the invitational leaflets were redesigned, and different members of the study team approached patients at different times. The resulting sample was notably homogenous, overrepresenting highly educated patients and patients with a higher interest in modern technologies. However, the comparison with prior research showed that similar themes were identified in our sample. Regarding future research in this area, we anticipate that researchers in other settings will face comparable recruitment issues. Thus, recruitment procedures should be planned carefully to also include participants who are typically underrepresented in research.

### Conclusions

The study presented in this paper led to the identification of necessary requirements for the development, testing, and validation of AI applications within a hospital-based generic infrastructure. A 4-layer model was developed, which will inform the next steps in the development of an AI innovation environment at our institution. Results from our study replicate previous findings from other contexts and will add to the emerging discussion on the use of routine medical data for the development of AI applications. Results will play a major role in the design and implementation of our infrastructure and processes of the AI innovation environment. In the context of the *pAItient* project, another qualitative study is planned at the end of the project to evaluate the AI innovation environment and to revisit the requirements presented in this study.

## Appendix

Multimedia Appendix 1 GRIPP2-Short Form. GRIPP2-SF: Guidance for Reporting Involvement of Patients and the Public—short form.

Multimedia Appendix 2 Translated interview guide.

Multimedia Appendix 3 Translated survey.

Multimedia Appendix 4 COREQ Checklist. COREQ: Consolidated Criteria for Reporting Qualitative Research.

Multimedia Appendix 5 Hardware and software used for artificial intelligence development.

## Abbreviations

AI: artificial intelligence
COREQ: Consolidated Criteria for Reporting Qualitative Research
DFKI: German Research Center for Artificial Intelligence
DKFZ: German Cancer Research Center
GDPR: General Data Protection Regulation
GRIPP2-SF: Guidance for Reporting Involvement of Patients and the Public—short form
IP: intellectual property
IT: information technology
pAItient: Protected Artificial Intelligence Innovation Environment for Patient Oriented Digital Health Solutions for developing, testing and evidence-based evaluation of clinical value
REDCap: Research Electronic Data Capture
UI: user interface

## References

[1] Meskó Bertalan, Hetényi Gergely, Győrffy Zsuzsanna (2018). Will artificial intelligence solve the human resource crisis in healthcare?. BMC Health Serv Res.

[2] van der Schaar Mihaela, Alaa Ahmed M, Floto Andres, Gimson Alexander, Scholtes Stefan, Wood Angela, McKinney Eoin, Jarrett Daniel, Lio Pietro, Ercole Ari (2021). How artificial intelligence and machine learning can help healthcare systems respond to COVID-19. Mach Learn.

[3] Noorbakhsh-Sabet Nariman, Zand Ramin, Zhang Yanfei, Abedi Vida (2019). Artificial Intelligence Transforms the Future of Health Care. Am J Med.

[4] Schwalbe Nina, Wahl Brian (2020). Artificial intelligence and the future of global health. Lancet.

[5] He Jianxing, Baxter Sally L, Xu Jie, Xu Jiming, Zhou Xingtao, Zhang Kang (2019). The practical implementation of artificial intelligence technologies in medicine. Nat Med.

[6] Yin Jiamin, Ngiam Kee Yuan, Teo Hock Hai (2021). Role of Artificial Intelligence Applications in Real-Life Clinical Practice: Systematic Review. J Med Internet Res.

[7] Cohen Adam B, Stump Lisa, Krumholz Harlan M, Cartiera Margaret, Jain Sanchita, Scott Sussman L, Hsiao Allen, Lindop Walter, Ying Anita Kuo, Kaul Rebecca L, Balcezak Thomas J, Tereffe Welela, Comerford Matthew, Jacoby Daniel, Navai Neema (2022). Aligning mission to digital health strategy in academic medical centers. NPJ Digit Med.

[8] Fritsch Sebastian J, Blankenheim Andrea, Wahl Alina, Hetfeld Petra, Maassen Oliver, Deffge Saskia, Kunze Julian, Rossaint Rolf, Riedel Morris, Marx Gernot, Bickenbach Johannes (2022). Attitudes and perception of artificial intelligence in healthcare: A cross-sectional survey among patients. Digit Health.

[9] De Nigri S, Craglia M, Nepelski D, Hradec J, Gómez-González E, Gomez E (2020). AI Watch : AI Uptake in Health and Healthcare 2020. JRC Publications Repository.

[10] Strohm Lea, Hehakaya Charisma, Ranschaert Erik R, Boon Wouter P C, Moors Ellen H M (2020). Implementation of artificial intelligence (AI) applications in radiology: hindering and facilitating factors. Eur Radiol.

[11] Weinert Lina, Müller Julia, Svensson Laura, Heinze Oliver (2022). Perspective of Information Technology Decision Makers on Factors Influencing Adoption and Implementation of Artificial Intelligence Technologies in 40 German Hospitals: Descriptive Analysis. JMIR Med Inform.

[12] Bonmatí Luis Martí, Miguel Ana, Suárez Amelia, Aznar Mario, Beregi Jean Paul, Fournier Laure, Neri Emanuele, Laghi Andrea, França Manuela, Sardanelli Francesco, Penzkofer Tobias, Lambin Phillipe, Blanquer Ignacio, Menzel Marion I, Seymour Karine, Figueiras Sergio, Krischak Katharina, Martínez Ricard, Mirsky Yisroel, Yang Guang, Alberich-Bayarri Ángel (2022). CHAIMELEON Project: Creation of a Pan-European Repository of Health Imaging Data for the Development of AI-Powered Cancer Management Tools. Front Oncol.

[13] Bukowski Mark, Farkas Robert, Beyan Oya, Moll Lorna, Hahn Horst, Kiessling Fabian, Schmitz-Rode Thomas (2020). Implementation of eHealth and AI integrated diagnostics with multidisciplinary digitized data: are we ready from an international perspective?. Eur Radiol.

[14] Shaw James, Rudzicz Frank, Jamieson Trevor, Goldfarb Avi (2019). Artificial Intelligence and the Implementation Challenge. J Med Internet Res.

[15] Aitken Mhairi, de St Jorre Jenna, Pagliari Claudia, Jepson Ruth, Cunningham-Burley Sarah (2016). Public responses to the sharing and linkage of health data for research purposes: a systematic review and thematic synthesis of qualitative studies. BMC Med Ethics.

[16] McCradden Melissa D, Baba Ami, Saha Ashirbani, Ahmad Sidra, Boparai Kanwar, Fadaiefard Pantea, Cusimano Michael D (2020). Ethical concerns around use of artificial intelligence in health care research from the perspective of patients with meningioma, caregivers and health care providers: a qualitative study. CMAJ Open.

[17] McCradden Melissa D, Sarker Tasmie, Paprica P Alison (2020). Conditionally positive: a qualitative study of public perceptions about using health data for artificial intelligence research. BMJ Open.

[18] Universitätsklinikum Heidelberg Medizinisches Datenintegrationszentrum (MEDIC). Institut für Medizinische Informatik.

[19] Wettstein Reto, Hund Hauke, Fegeler Christian, Heinze Oliver (2021). Data Sharing in Distributed Architectures - Concept and Implementation in HiGHmed. Stud Health Technol Inform.

[20] Weinert Lina, Klass Maximilian, Schneider Gerd, Heinze Oliver (2022). Exploring Stakeholder Requirements to Enable the Research and Development of Artificial Intelligence Algorithms in a Hospital-Based Generic Infrastructure: Protocol for a Multistep Mixed Methods Study. JMIR Res Protoc.

[21] Staniszewska S, Brett J, Simera I, Seers K, Mockford C, Goodlad S, Altman D G, Moher D, Barber R, Denegri S, Entwistle A, Littlejohns P, Morris C, Suleman R, Thomas V, Tysall C (2017). GRIPP2 reporting checklists: tools to improve reporting of patient and public involvement in research. Res Involv Engagem.

[22] Kamradt Martina, Poß-Doering Regina, Szecsenyi Joachim (2022). Exploring Physician Perspectives on Using Real-world Care Data for the Development of Artificial Intelligence-Based Technologies in Health Care: Qualitative Study. JMIR Form Res.

[23] Harris Paul A, Taylor Robert, Thielke Robert, Payne Jonathon, Gonzalez Nathaniel, Conde Jose G (2009). Research electronic data capture (REDCap)--a metadata-driven methodology and workflow process for providing translational research informatics support. J Biomed Inform.

[24] Harris Paul A, Taylor Robert, Minor Brenda L, Elliott Veida, Fernandez Michelle, O'Neal Lindsay, McLeod Laura, Delacqua Giovanni, Delacqua Francesco, Kirby Jacqueline, Duda Stephany N, REDCap Consortium (2019). The REDCap consortium: Building an international community of software platform partners. J Biomed Inform.

[25] Tong Allison, Sainsbury Peter, Craig Jonathan (2007). Consolidated criteria for reporting qualitative research (COREQ): a 32-item checklist for interviews and focus groups. Int J Qual Health Care.

[26] Braun V, Clarke V (2006). Using thematic analysis in psychology. Qualitative Research in Psychology.

[27] European Parliament, Council of the European Union (2016). Regulation 2016/679: Protection of natural persons with regard to the processing of personal data and on the free movement of such data, and repealing Directive 95/46/EC (General Data Protection Regulation). EU Monitor.

[28] Hamon R, Junklewitz H, Malgieri G, Hert PD, Beslay L, Sanchez I (2021). Impossible explanations?: Beyond explainable AI in the GDPR from a COVID-19 use case scenario. FAccT '21: Proceedings of the 2021 ACM Conference on Fairness, Accountability, and Transparency.

[29] Kaminski ME (2019). The Right to Explanation, Explained. Berkeley Tech Law J.

[30] Willison Donald J, Swinton Marilyn, Schwartz Lisa, Abelson Julia, Charles Cathy, Northrup David, Cheng Ji, Thabane Lehana (2008). Alternatives to project-specific consent for access to personal information for health research: insights from a public dialogue. BMC Med Ethics.

[31] Tosoni Sarah, Voruganti Indu, Lajkosz Katherine, Habal Flavio, Murphy Patricia, Wong Rebecca K S, Willison Donald, Virtanen Carl, Heesters Ann, Liu Fei-Fei (2021). The use of personal health information outside the circle of care: consent preferences of patients from an academic health care institution. BMC Med Ethics.

[32] Paprica P Alison, de Melo Magda Nunes, Schull Michael J (2019). Social licence and the general public's attitudes toward research based on linked administrative health data: a qualitative study. CMAJ Open.

[33] Kim Jihoon, Kim Hyeoneui, Bell Elizabeth, Bath Tyler, Paul Paulina, Pham Anh, Jiang Xiaoqian, Zheng Kai, Ohno-Machado Lucila (2019). Patient Perspectives About Decisions to Share Medical Data and Biospecimens for Research. JAMA Netw Open.

[34] Reddy Sandeep, Allan Sonia, Coghlan Simon, Cooper Paul (2020). A governance model for the application of AI in health care. J Am Med Inform Assoc.

[35] Ploug Thomas, Sundby Anna, Moeslund Thomas B, Holm Søren (2021). Population Preferences for Performance and Explainability of Artificial Intelligence in Health Care: Choice-Based Conjoint Survey. J Med Internet Res.

[36] Keusch F (2015). Why do people participate in Web surveys? Applying survey participation theory to Internet survey data collection. Manag Rev Q.

