# Supplementary material for: Exploring Stakeholder Requirements to Enable Research and Development of Artificial Intelligence Algorithms in a Hospital-Based Generic Infrastructure: Results of a Multistep Mixed Methods Study
Source: JMIR Form Res. 2023 Apr 18;7:e43958. doi: 10.2196/43958 (PMC10155093; doi:10.2196/43958)
Supplement: Multimedia Appendix 1 [file formative_v7i1e43958_app1.pdf]

Appendix: GRIPP2 Short-form checklist

Section and topic	Item
**1: Aim**<br>Report the aim of the study	To explore stakeholders' requirements for developing AI in partnership with an academic hospital and granting AI experts access to anonymized personal health data.
**2: Methods**<br>Provide a clear description of the methods used for PPI in the study	Researchers and employees from stakeholder organizations were invited to participate in semi-structured interviews. Questionnaires were developed based on the participants' answers and distributed among the stakeholders' organizations. Additionally, patients and physicians were interviewed as well.
**3: Results**<br>Outcomes—Report the results of PPI in the study, including both positive and negative outcomes	Stakeholders informed the study team about necessary requirements for the development of AI and the use of their data for health research.
**4: Discussion**<br>Outcomes—Comment on the extent to which PPI influenced the study overall. Describe positive and negative effects	The study led to the identification of necessary requirements for the development, testing, and validation of AI applications within a hospital-based generic infrastructure. A model was developed, which will inform the next steps in the development of an AI Innovation Environment at our institution. Requirements derived from the PPI interviews influenced every aspect of the newly developed model.
**5: Reflection**<br>Critical perspective—Comment critically on the study, reflecting on the things that went well and those that did not, so others can learn from this experience	PPI was fruitful and important insights were collected. However, recruitment for study participation was very challenging. This could influence the results and requirements identified.<br>In a future replication of a similar study design, it could be helpful to involve stakeholders at an earlier time so the study design itself (including recruitment methods) could be co-developed.

GRIPP2-SF adapted from: Staniszewska S, Brett J, Simera I, Seers K, Mockford C, Goodlad S, Tysall C. GRIPP2 reporting checklists: tools to improve reporting of patient and public involvement in research. Research Involvement and Engagement. 2017 3 (1)
