# Supplementary material for: Exploring Stakeholder Requirements to Enable Research and Development of Artificial Intelligence Algorithms in a Hospital-Based Generic Infrastructure: Results of a Multistep Mixed Methods Study
Source: JMIR Form Res. 2023 Apr 18;7:e43958. doi: 10.2196/43958 (PMC10155093; doi:10.2196/43958)
Supplement: Multimedia Appendix 3 [file formative_v7i1e43958_app3.pdf]

Appendix 3: Translated surveys

How much experience do you have with projects regarding AI in medicine and health care?

- A lot of experience
- Some experience
- Little experience
- No experience

1. **Hardware and software**

Do you use the following hardware (components) for the development/testing of AI tools?

Biomedical AI researchers	AI research institution	Software developing companies
GPU (Cluster)	GPU	(high end) consumer
Cloud nodes	NVIDIA components	
NVIDIA DGX		

Do you use any other hardware (components) that were not listed above?

____

Do you use the following software for the development/testing of AI tools?

Biomedical AI researchers	AI research institution	Software developing companies
PyCharm	Python infrastructures/libraries	
PyTorch	PyTorch	PyTorch
Jupyter Notebooks		
TensorFlow	TensorFlow	TensorFlow
Kapaana		
Caffe2		
Corti		
Azure		
Communication Message Broker (z.B. Kafka)		

Self-developed toolkits		
	OpenCV	
		Onyx

Do you use any other hardware (components) that were not listed above?

____

2. Goals

The following list contains possible goals motivating you or your organization. Please tell us how important these goals are for you by moving the slider.

Biomedical AI researchers	AI research institution	Software developing companies
Reaching research goals (e.g., publications, presentations at conferences)	Reaching research goals (e.g., publications, presentations at conferences)	
Founding of new companies/spin-off companies		
Development of patents		
Benefit for patients and patient care	Benefit for patients and patient care	
		Successful product development
		patents

What other goals are important for you and your institution?

____

3. Types of data

What data or what kind of data do you typically need for your projects? Please check the according boxes.

Biomedical AI researchers	AI research institution	Software developing companies
text with context information (e.g., for extracting information from text)	text with context information (e.g., for extracting information from text)	text with context information (e.g., for extracting information from text)
Image data (e.g., for image processing)	Image data (e.g., for image processing)	Image data (e.g., for image processing)
external data/sources (e.g., SNOMED, ICD-10)	External data/sources (e.g., SNOMED, ICD-10)	External data/sources (e.g., SNOMED, ICD-10)
Structured EHR data		
Unstructured EHR data		
Data from non-medical peripheral sensors (e.g., OR doors)		
Ground truth/characterized/annotated data		
External sources (e.g., local treatment guidelines)		
Data from medical instruments (e.g., intraoperative endoscopy)		

What other kinds of data do you typically work with?

____

4. Heidelberg University Hospital as a data provider

Do you have experience with the Heidelberg University Hospital as a data provider?

☐ **Yes**

☐ **No**

If yes:

To what extent do you agree with the following potential advantages of the Heidelberg University Hospital as a data provider? Please rate your agreement with these potential advantages on a scale from 0 (= I do not agree at all) to 5 (= completely agree).

Biomedical AI researchers	Software developing companies
Data from a "real environment"	
Personal contact with future users of the developed AI tools	
Simplified identification of potential use cases	
Extensive technical facilities	
Scaling effects	
Many potential clinical partners	
High degree of professionalisation	
Hospital takes on administrative tasks	
Availability of contact person in case of queries in the data	
	Large amount of data available
	Expertise of clinical partners
	Routine care follows medical guidelines
	Hospital staff can take time for research activities

What further potential positive aspects have you encountered?

____

AI research institutions:

Working with data from a university hospital, what advantages did you observe for your project/your activities? Were there any positive aspects?

To what extent do you agree with the following potential disadvantages of the Heidelberg University Hospital as a data provider? Please rate your agreement with these potential disadvantages on a scale from 0 (= I do not agree at all) to 5 (= completely agree).

Biomedical AI researchers	Software developing companies
Complex legal requirements concerning data privacy and other regulations	
Complex bureaucracy	
Diversity and number of contact persons	
Requirement to work within research studies	
Legal and regulatory fears among (hospital) administrative staff	
Overfitting of a developed algorithm to one hospital	
Decision-makers not competent to understand complex innovations and their implications	Decision-makers not competent to understand complex innovations and their implications
Discussions about intellectual property	
Tools are too complex	
Launching new projects is cumbersome	
Data quality not adequate	
	Slow (administrative) processes
	Involvement of a large number of decision-makers

What further potential negative aspects have you encountered?

____

AI research institutions:

Working with data from a university hospital, what disadvantages did you observe for your project/your activities? Were there any negative aspects?

5. **Potential areas for support**

The following list contains possible support measures a university hospital could offer as a data source. Please tell us how helpful you think these measures would be for your work by rating these potential areas for support on a scale from 0 (= I do not agree at all) to 5 (= completely agree).

Biomedical AI researchers	AI research institution	Software developing companies
Simplified, standardized process for data privacy processes		Simplified, standardized process for data privacy processes
Establish or simplify contact to clinical users		Establish or simplify contact to clinical users
Provide insight into clinical processes		
Platform offering an overview of previously developed algorithms		
Platform offering an overview of available data		
Platform offering an overview of potential partners for cooperation		
Data from medical instruments (e.g., intraoperative endoscopy)		
Transparent communication about character and quality of available data		
Provision of annotated data		
Enabling of federated learning		
Combination of EHR and research data base		

More opportunities to apply or test developed tools in a hospital environment		
	Finding research questions/medical use cases in a collective approach with clinicians	
	Protection from and clarification of liability issues	

From your point of view, are there any other supportive measures a university hospital should offer? If yes, please name them.

---

6. Requirements

In the following list you will find possible prerequisites that could be necessary for collaborating with a university hospital. Please tell us how important the fulfillment of these prerequisites is for you by rating these prerequisites on a scale from 0 (= I do not agree at all) to 5 (= completely agree).

AI research institution	Software developing companies
guaranteed involvement of clinical experts	
use case in place and defined	
	The topic of IP has to be regulated within in defined requirements

In your opinion, are there any other prerequisites the Heidelberg University Hospital should fulfill? If yes, please tell us.

---

7. Conclusion

Concerning this topic, what other requirements or recommendations do you have?
