# Supplementary material for: Exploring Stakeholder Requirements to Enable Research and Development of Artificial Intelligence Algorithms in a Hospital-Based Generic Infrastructure: Results of a Multistep Mixed Methods Study
Source: JMIR Form Res. 2023 Apr 18;7:e43958. doi: 10.2196/43958 (PMC10155093; doi:10.2196/43958)
Supplement: Multimedia Appendix 5 [file formative_v7i1e43958_app5.pdf]

Appendix: Software and hardware used for AI development

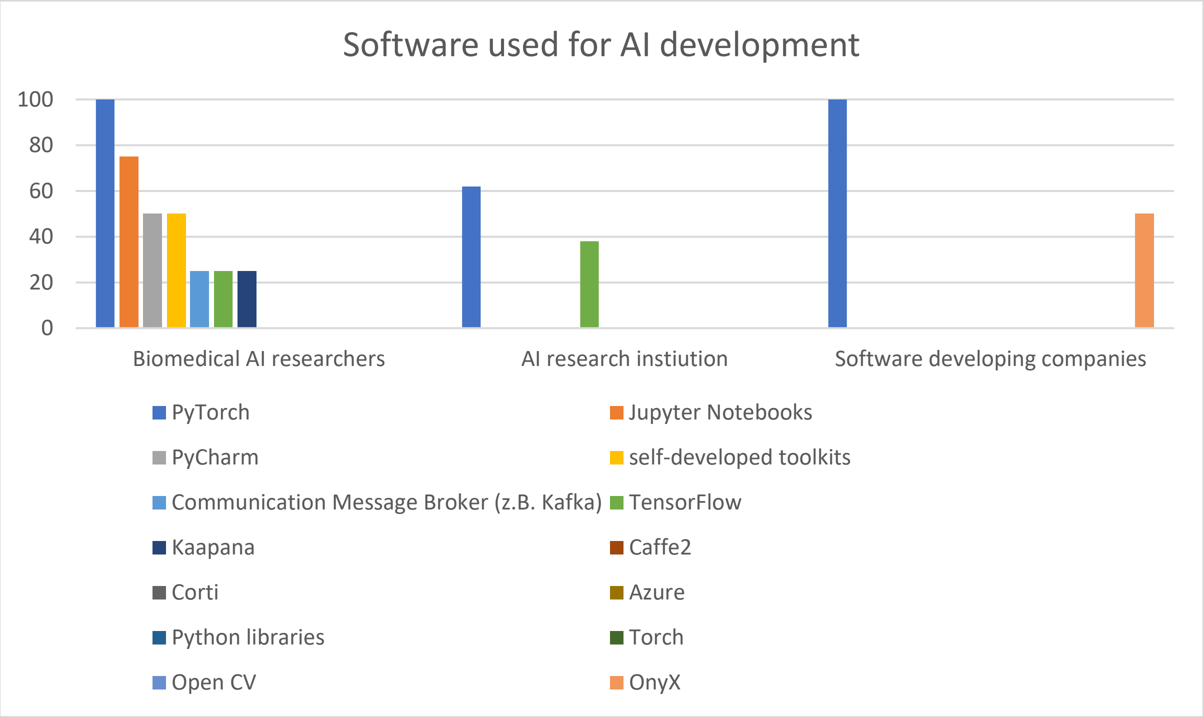

Fig. 1 – Software used for the development of AI tools

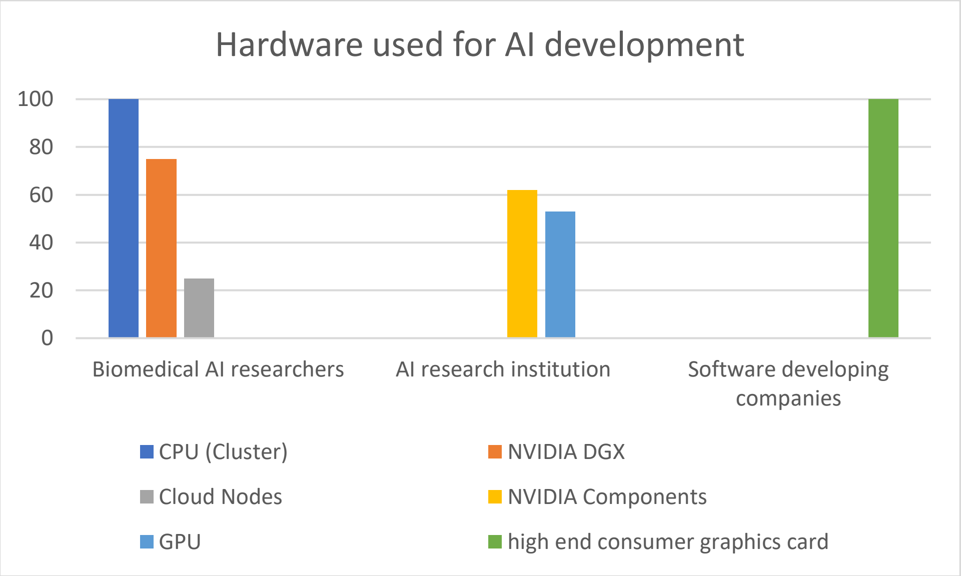

Fig.2 – Hardware used for the development of AI tools
